# Delayed versus early umbilical cord clamping for near-term infants born to preeclamptic mothers; a randomized controlled trial

**DOI:** 10.1186/s12884-022-04831-8

**Published:** 2022-06-25

**Authors:** Ahmed Rashwan, Ashraf Eldaly, Ahmed El-Harty, Moutaz Elsherbini, Mazen Abdel-Rasheed, Marwa M. Eid

**Affiliations:** 1 Obstetrics and Gynaecology Department, Faculty of Medicine, Cairo University, Cairo, Egypt; 2 Reproductive Health Research Department, National Research Centre, 33 El-Buhouth St, Dokki, Cairo, 12622 Egypt

**Keywords:** Umbilical cord clamping, Preeclampsia, NICU, Neonatal jaundice, Neonatal haemoglobin

## Abstract

**Objective:**

This study aims to assess delayed versus early umbilical cord clamping in preeclamptic mothers undergoing scheduled caesarean delivery regarding the maternal intra-operative blood loss and neonatal outcomes.

**Methods:**

A clinical trial was conducted on 62 near-term preeclamptic mothers (36-38^+6^ weeks) who were planned for caesarean delivery. They were randomly assigned into two groups. The first group was the early cord clamping (ECC) group (*n*= 31), in which clamping the umbilical cord was within 15 seconds, while the second group was the delayed cord clamping (DCC) group (*n*= 31), in which clamping the umbilical cord was at 60 seconds. All patients were assessed for intra-operative blood loss and incidence of primary postpartum haemorrhage (PPH). Otherwise, all neonates were assessed for APGAR scores, the need for the neonatal intensive care unit (NICU) admission due to jaundice, and blood tests (haemoglobin, haematocrit. and serum bilirubin).

**Results:**

There was not any significant difference between the two groups regarding the maternal estimated blood loss (*P*=0.673), the rates of PPH (*P*=0.1), post-delivery haemoglobin (*P*=0.154), and haematocrit values (*P*=0.092). Neonatal outcomes also were showing no significant difference regarding APGAR scores at the first minute (*P*=1) and after 5 minutes (*P*=0.114), day 1 serum bilirubin (*P*=0.561), day 3 serum bilirubin (*P*=0.676), and the rate of NICU admission (*P*=0.671). However, haemoglobin and haematocrit values were significantly higher in the DCC group than in the ECC group (*P*<0.001).

**Conclusion:**

There is no significant difference between DCC and ECC regarding maternal blood loss. However, DCC has the advantage of significantly higher neonatal haemoglobin.

**Trial registration:**

It was first registered at ClinicalTrials.gov on 10/12/2019 with registration number NCT04193345.

## Introduction

Delayed umbilical cord clamping has shown enormous health advantages for both preterm and term infants, as demonstrated in several randomized controlled trials and meta-analyses [1, 2]. In term infants, it was found that delayed cord clamping (DCC) increased neonatal haemoglobin and ferritin levels stores out to 4 months with higher myelin content out to 12 months [3]. DCC in preterm infants also showed many significant benefits, including establishing better red blood cell volume, decreasing the need for blood transfusion, improving the transitional circulation, and lowering the incidence of intraventricular haemorrhage and necrotizing enterocolitis [4]. As a result, the American College of Obstetricians and Gynaecologists (ACOG) recommends DCC for at least 30–60 seconds after birth in both preterm and term newborns [5].

In patients undergoing caesarean section, the average blood loss is at least double the vaginal deliveries [6, 7]. This blood loss may increase with the delay in the uterine incision closure in DCC [8–10]. However, a systematic review on DCC at term presents no significant differences in the maternal blood loss, although those included studies were done on low-risk patients expected to deliver vaginally [1].

In DCC, there is an increase of about 20-30% in infants’ blood volume with a 50% increase in red blood cell volume [11]. In the latest Cochrane database review on term infants, it has been found that there were no differences in the infants’ outcomes between early cord clamping (ECC) and DCC regarding neonatal morbidities such as neonatal intensive care unit (NICU) admission, APGAR scores <7 at 5 minutes, or clinical jaundice [1].

Preeclampsia complications are around 10-15% of all pregnancies and are usually associated with several foetal and neonatal complications related to prematurity and uteroplacental insufficiency and are causing a compromised foetal blood flow [12]. The risks of polycythaemia and thrombocytopenia were higher in neonates born to mothers with hypertensive pregnancy disorders than in the general population [13]. Additionally, changes that occur in a normal, uncomplicated pregnancy, including hyperlipidaemia, neutrophilic leucocytosis, and hypofibrinolytic changes, were found to be enhanced in preeclampsia and together with the presence of placental abnormalities resulting in both foetal and neonatal complications [14, 15].

Newborns of preeclamptic mothers are at risk of many complications; however, no study has thoroughly investigated the effect of DCC on maternal blood loss during caesarean delivery and the neonatal outcomes in these pregnancies. Therefore, it was the focus of our study

## Methods

Following the CONSORT guidelines, a randomized clinical trial was conducted in Kasr El-Ainy Hospital (Obstetrics and Gynaecology Department, Faculty of Medicine, Cairo University) from January 2020 to May 2021 after approval of the Medical Ethical Committee. It was first registered at ClinicalTrials.gov on 10/12/2019 with registration number NCT04193345.

The study included 62 pregnant women diagnosed with preeclampsia at near-term (36-38^+6^ weeks, i.e., late-preterm and early-term). All study cases had been assigned for lower segment caesarean section (LSCS) under spinal anaesthesia. According to ACOG, preeclampsia was diagnosed with new-onset hypertension after 20 weeks of gestation accompanied by either new-onset proteinuria or new-onset of any of the following; thrombocytopenia, renal insufficiency, impaired liver function, pulmonary oedema, or headache unresponsive to medication [16].

Inclusion criteria were maternal age 20-40 years, gestational age ≥36 weeks, and singleton living healthy foetus. Women who had intrapartum surgical complications such as uterine artery injury or lower segment extension, IUFD, or cases with medical disorders such as severe anaemia or diabetes mellitus were excluded. Women with abnormal placentation, placenta abruption, liquor abnormalities, or anomalous foetuses were also excluded.

Informed consent was obtained from all patients after explaining the aim of the study. For all participants, full history was taken, followed by a complete physical examination and routine obstetric ultrasound to confirm the eligibility of the current pregnancy to participate in the study, as well as to confirm the gestational age. The routine preoperative laboratory tests were performed, including complete blood count, liver and kidney function tests, prothrombin time and prothrombin concentration.

On the day of the scheduled caesarean delivery, participants were randomly assigned using computer-generated random numbers into two equal groups; the ECC group (*n*= 31) in whom the umbilical cord was clamped within 15 seconds and the DCC group (*n*= 31) in whom the umbilical cord was clamped at 60 seconds. Caesarean sections were done under spinal anaesthesia by an experienced obstetrician, while recording the time between the delivery and cord clamping was the responsibility of a research staff member who attended all deliveries. For the DCC group, the research staff member recorded 60 seconds before asking the obstetrician to clamp the umbilical cord. During this period, their neonates were placed on the sterile drapes on the mother’s legs at the level of the placenta until cord clamping was performed.

The attending neonatologist assessed all neonates in both groups for APGAR scores, jaundice, and the need for neonatal ICU admission. Neonatal haemoglobin and haematocrit were done 4 hours after delivery and then repeated after 24 hours. Serum bilirubin was done 12 hours after delivery and repeated on day 3 for follow-up.

The number of the operative towels and the blood volume in the suction unit were recorded. The maternal complete blood count (CBC) test was repeated after 12 hours. All mothers were observed for primary postpartum haemorrhage (PPH) and the need for blood transfusion for the first 24 hours. The estimated blood loss (EBL) was calculated by the following formula:

$$\mathrm{EBL}=\mathrm{EBV}\times \frac{\mathrm{Preoperative}\kern0.34em \mathrm{hematocrit}\hbox{-} \mathrm{Postoperative}\kern0.34em \mathrm{hematocrit}}{\mathrm{Preoperative}\kern0.34em \mathrm{hematocrit}},$$

where EBV is the estimated blood volume of the patient in mL= weight in kg × 85 [17].

The primary outcome was comparing the effect of DCC versus the ECC on maternal intraoperative blood loss, while the secondary outcomes were comparing both groups regarding the neonatal outcomes and the incidence of postpartum haemorrhage.

### Sample size calculation

The sample size for each group of 28 achieves 70% power to detect a difference of 0.04 between the null hypothesis that both group means are 0.61 and the alternative hypothesis that the mean of group 2 is 0.57 with estimated group standard deviations of 0.05 and 0.07 and with a significance level (alpha) of 0.05 using a two-sided two-sample t-test [18]. The sample size increased by 10% to be 31 for each group to allow for dropouts.

### Statistical methods

Data were coded and entered using the statistical package for the Social Sciences (SPSS) version 26 (IBM Corp., Armonk, NY, USA). Data were summarized using mean and standard deviation for continuous quantitative variables; and frequencies (number of cases) and relative frequencies (percentages) for categorical variables. The independent samples t-test was used to compare groups regarding the numerical data. For comparing categorical data, a Chi-square test was performed, but Fisher’s exact test was used instead when the expected frequency was less than 5. *P* values less than 0.05 were considered statistically significant.

## Results

**Fig. 1:**
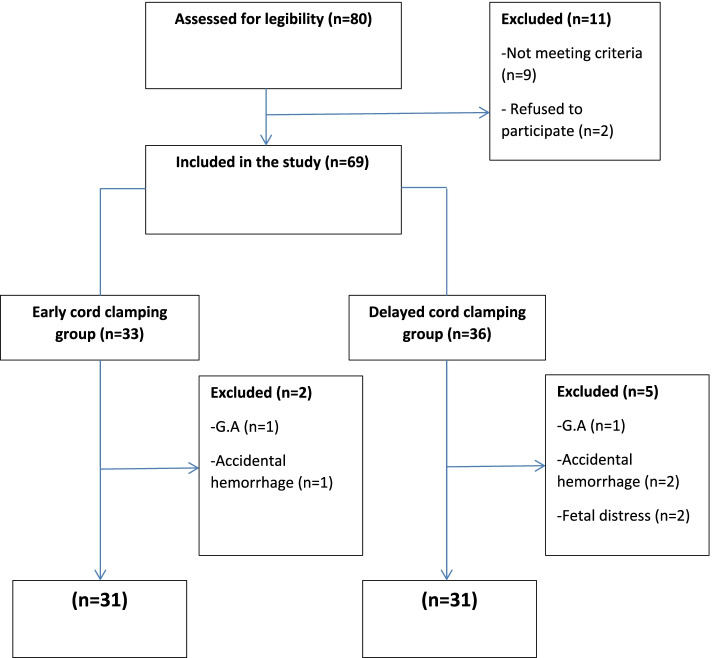
Flow diagram of patients in the study

Sixty-two pregnant women were diagnosed with preeclampsia, and being candidates for LSCS under spinal anaesthesia were finally included and followed up in this study. The flow of patients was summarised in Fig. 1. As previously defined, the patients were randomly and equally assigned into two groups; ECC (*n*= 31) and DCC (*n*= 31). Patients’ baseline clinical characteristics and demographic data did not show any significant difference between these two groups Table 1.

**Table 1:** Maternal demographic data and baseline clinical characteristics

	ECC(n=31)	DCC(n=31)	*p* value
**Maternal age**	30.71 ± 5.72	30.61 ± 4.62	0.942
**Gestational age (weeks)**	36.94 ± 0.63	36.74 ± 0.68	0.253
**BMI (kg/m2)**	34.71 ± 5.39	34.55 ± 5.00	0.903
**Parity**			
**- Primigravida**	12 (38.7%)	11 (35.5%)	0.793
**- Multigravida**	19 (61.29%)	20 (64.52%)	
**Mean systolic BP (mmHg)**	176.90 ± 16.13	179.74 ± 16.42	0.495
**Mean diastolic BP (mmHg)**	103.74 ± 8.33	105.58 ± 7.64	0.369
**Initial HB (g/dl)**	11.82 ± 0.98	11.25 ± 1.33	0.059
**Initial HCT (%)**	34.25 ± 2.89	32.65 ± 4.38	0.094
**Platelets count**	207.68 ± 56.18	204.84 ± 55.8	0.842
**Proteinuria +1**	10 (32.26%)	9 (29.03%)	0.937
**Proteinuria +2**	15 (48.39%)	15 (48.39%)	
**Proteinuria +3**	6 (19.35%)	7 (22.58%)	

As shown in Table 2, there was no noteworthy difference in maternal estimated blood loss between the two groups at the time of caesarean section (*P*=0.673) nor the rates of PPH during the first 24 hours (*P*=0.1). Moreover, biochemical examination revealed no significant difference between both groups regarding the post-delivery haemoglobin (*P*=0.154) and post-delivery haematocrit (*P*=0.092).

**Table 2:** Maternal outcomes in Caesarean section

	ECC (***n***=31)	DCC (***n***=31)	***p*** value
**Number of soaked towels**	5.32 ± 1.87	5.13 ± 2.33	0.720
**Blood loss in suction apparatus (ml)**	566.13 ± 126.09	606.45 ± 105.47	0.177
**Estimated blood loss (ml)**	756.29 ± 194.72	784.97 ± 322.39	0.673
**Postpartum Hb (g/dl)**	10.59 ± 1.10	10.18 ± 1.12	0.154
**Postpartum HCT (%)**	30.72 ± 2.90	29.20 ± 4.05	0.092
**Incidence of postpartum haemorrhage**	2 (6.45%)	3 (9.68%)	0.100

When neonatal outcomes were analyzed, the haemoglobin and haematocrit values were significantly higher within the DCC group during the first day if compared to the ECC group (18.89 ± 1.55 versus 17.01 ± 1.70, *P*<0.001 for haemoglobin and 56.05 ± 3.91 versus 50.44 ± 4.34, *P*<0.001 for haematocrit) as well as during the second day (17.90 ± 1.08 versus 16.29 ± 1.41, *P*<0.001 for haemoglobin and 53.09 ± 3.64 versus 47.85 ± 3.77, *P*<0.001 for haematocrit). On the contrary, there was no significant difference between both groups regarding APGAR scores at the first minute (*P*=1) and after 5 minutes (*P*=0.114), day 1 serum bilirubin (*P*=0.561), and day 3 serum bilirubin (*P*=0.676). Additionally, there was no significant difference between the two groups regarding the rate of NICU admission (6.45% in the ECC group versus 12.9% in the DCC group, *P*=0.671), as shown in Table 3.

**Table 3:** Neonatal clinical characteristics

	ECC (***n***=31)	DCC (***n***=31)	***p*** value
**Gestational age**			
- **36 weeks**	7 (22.58%)	12 (38.71%)	0.387
- **37 weeks**	19 (61.29%)	15 (48.39%)	
- **38 weeks**	5 (16.13%)	4 (12.90%)	
**Neonatal weight (gm)**	2922.26 ± 118.74	2882.58 ± 232.25	0.401
**APGAR at the first minute.**	5.45 ± 0.85	5.45 ± 0.57	1.000
**APGAR after 5 minutes.**	8.55 ± 0.51	8.32 ± 0.60	0.114
**Neonatal day 1 Hb (g/dl)**	17.01 ± 1.70	18.89 ± 1.55	<0.001
**Neonatal day 2 Hb (g/dl)**	16.29 ± 1.41	17.90 ± 1.08	<0.001
**Neonatal day 1 HCT (%)**	50.44 ± 4.34	56.05 ± 3.91	<0.001
**Neonatal day 2 HCT (%)**	47.85 ± 3.77	53.09 ± 3.64	<0.001
**Neonatal day 1 bilirubin (mg/dl)**	2.37 ± 0.68	2.26 ± 0.73	0.561
**Neonatal day 3 bilirubin (mg/dl)**	9.91 ± 1.89	10.14 ± 2.48	0.676
**NICU admission (%)**	2 (6.45%)	4 (12.90%)	0.671

The results are described in the form of mean ± standard deviation (SD) and frequency (percentage), *p* < 0.05 is considered statistically significant

## Discussion

In our trial, the maternal and neonatal outcomes of DCC for 60 seconds during caesarean delivery for near-term pregnancies (36-38^+6^ weeks) complicated with preeclampsia were studied. To our knowledge, our study is the first randomized trial evaluating DCC versus ECC in caesarean delivery in pregnancies complicated by preeclampsia.

Traditionally, there were some concerns about the risk of increasing maternal blood loss due to prolongation of the operative time as a consequence of delayed wound closure in the DCC group, and that may predispose to uterine atony [8–10]. Therefore, a concern about the benefits of DCC during caesarean delivery was present, plus there were some barriers to its application [19]. However, recent studies revealed no significant increase in morbidity in terms of EBL or post-caesarean drop in maternal haemoglobin and haematocrit between ECC and DCC techniques [20]. In our study, we found that the DCC in near-term pregnancies (36-38^+6^ weeks) complicated with preeclampsia did not result in increased maternal blood loss compared to those who had ECC.

Similar to our study, a study on 39 women scheduled for caesarean delivery had DCC for 90 to 120 seconds and were compared with 112 historical controls who had immediate cord clamping. The authors found no difference in maternal postoperative/preoperative haemoglobin levels [21]. *Ruangkit et al.* paradoxically reported the mean EBL was higher in the ECC group, which is mostly not related to the technique in the umbilical cord management but most likely due to the significantly higher rate of caesarean section delivery in the ECC group compared to the DCC group in their study [22].

A particular strength in this study was the objective way of assessing blood loss in means of the preoperative versus the postoperative change in the haemoglobin and haematocrit levels. There is an agreement with Purisch et al. report of no significant difference in maternal blood loss using objective assessment methods, such as postoperative haemoglobin levels. They also found no substantial increase in uterotonic therapy or blood transfusion rates with caesarean delivery [23]. On the other hand, *Rhoades et al.* compared outcomes between the ECC versus DCC at term in a group of 196 women delivered by caesarean section. They found increased postpartum haemorrhage (> 1000 cc) in caesarean deliveries [24]. However, this is most probably related to the subjective ways of assessing blood loss.

A noteworthy increase in haemoglobin levels in the neonates of the DCC group at 4 then at 24 hours of life with no significant increase in jaundice is reported here in our study. Previous studies have assumed the disadvantage of ECC is losing the benefits of DCC. *Purisch et al.* reported a significant increase in neonatal haemoglobin levels at 24 to 72 hours of life could be achieved by the DCC technique in scheduled caesarean deliveries [23]. In addition, *Mercer et al.* reported an increase in neonatal haematocrit and haemoglobin at 24 to 48 hours with no increase in symptomatic polycythaemia, jaundice, or other adverse effects [25]. Our study also is in agreement with *McDonald et al.* (mean difference, 1.5 g/dL [95% CI, 1.21-1.78]), who also reported a significant increase in neonatal haemoglobin levels in the DCC group. In addition to the improved haematological status of neonates with DCC, no difference was reported in the recent studies between ECC and DCC regarding neonatal jaundice and phototherapy requirements [26, 27].

To the best of our knowledge, our study is the first to report maternal and neonatal outcomes with the different umbilical cord management techniques in preeclamptic patients. Another point of strength in our study was using objective, accurate methods of assessing the blood loss, unlike several studies using subjective methods to assess blood loss as postpartum haemorrhage [28]. Limitations of this study included the small sample size, which went back to patient selection based on near-term preeclampsia patients with singleton pregnancies scheduled for elective caesarean section. Therefore, our results may not be generalized to other situations with an emergency preterm delivery or cases who delivered vaginally. These cases warrant broader clinical trials to assess the effect of DCC as well. Another limitation is that longer durations of DCC have not been assessed in our trial as recommended in other previous studies [29]. Neonatal benefits were demonstrated with DCC in term neonates after 3 minutes and in preterm neonates after 30-180 seconds [30].

The American College of Obstetricians and Gynaecologists recommends the universal application of delayed umbilical cord clamping for infants for at least 30–60 seconds [5]. This made the DCC more widely used by obstetricians, but high-risk patients still need more randomized controlled trials to ensure safe maternal and neonatal outcomes. This study supports a basis that DCC can be safely used in preeclamptic mothers without unfavourable maternal outcomes.

## Conclusion

Among near-term (36-38^+6^ weeks) singleton pregnant preeclamptic mothers scheduled for caesarean delivery, there was no significant difference in maternal blood loss between ECC and DCC groups. However, neonatal haemoglobin and haematocrit were significantly higher with delayed umbilical cord clamping during the first and second days after delivery.

## Data Availability

The data that support the findings of this study are available from Kasr El-Ainy Hospital, but restrictions apply to the availability of these data, which were used under license for the current study, and so are not publicly available. Data are, however, available from the authors upon reasonable request and with permission of Kasr El-Ainy Hospital.
